# Sustained Effects of Acupuncture Stimulation Investigated with Centrality Mapping Analysis

**DOI:** 10.3389/fnhum.2016.00510

**Published:** 2016-10-18

**Authors:** Xiangyu Long, Wenjing Huang, Vitaly Napadow, Fanrong Liang, Burkhard Pleger, Arno Villringer, Claudia M. Witt, Till Nierhaus, Daniel Pach

**Affiliations:** ^1^Department of Neurology, Max Planck Institute for Human Cognitive and Brain SciencesLeipzig, Germany; ^2^Institute for Social Medicine, Epidemiology, and Health Economics, Charité – Universitätsmedizin BerlinBerlin, Germany; ^3^Acupuncture and Tuina School, The 3rd Teaching Hospital, Chengdu University of Traditional Chinese MedicineChengdu, China; ^4^Athinoula A. Martinos Center for Biomedical Imaging, Department of Radiology, Massachusetts General Hospital, CharlestownMA, USA; ^5^Department of Radiology, Logan University, ChesterfieldMO, USA; ^6^The Mind-Brain Institute at Berlin School of Mind and Brain, Charité and Humboldt-UniversitätBerlin, Germany; ^7^Institute for Complementary and Integrative Medicine, University of Zurich and University Hospital ZurichZurich, Switzerland; ^8^Neurocomputation and Neuroimaging Unit, Department of Education and Psychology, Freie Universität BerlinBerlin, Germany

**Keywords:** resting-state fMRI, acupuncture, functional connectivity, centrality, pain

## Abstract

Acupuncture can have instant and sustained effects, however, its mechanisms of action are still unclear. Here, we investigated the sustained effect of acupuncture by evaluating centrality changes in resting-state functional magnetic resonance imaging after manually stimulating the acupuncture point ST36 at the lower leg or two control point locations (CP1 same dermatome, CP2 different dermatome). Data from a previously published experiment evaluating instant BOLD effects and S2-seed-based resting state connectivity was re-analyzed using eigenvector centrality mapping and degree centrality mapping. These data-driven methods might add new insights into sustained acupuncture effects on both global and local inter-region connectivity (centrality) by evaluating the summary of connections of every voxel. We found higher centrality in parahippocampal gyrus and middle temporal gyrus after ST36 stimulation in comparison to the two control points. These regions are positively correlated to major hubs of the default mode network, which might be the primary network affected by chronic pain. The stronger integration of both regions within the whole-brain connectome after stimulation of ST36 might be a potential contributor to pain modulation by acupuncture. These findings highlight centrality mapping as a valuable analysis for future imaging studies investigating clinically relevant outcomes associated with physiological response to acupuncture stimulation. Clinical trial registration: NCT01079689, ClinicalTrials.gov.

## Introduction

The time-variant characteristic of acupuncture includes instant effects as well as sustained effects (Li et al., 2007), which may contribute both to a successful treatment. Clinical reports (Vickers, 2004; Melchart et al., 2005; Li et al., 2012; Ma et al., 2012) and systematic reviews (White et al., 2007; Linde et al., 2009) have provided evidence that the acupuncture effect can last far beyond its application. Also, studies using animal models found that there are sustained effects which might accumulate at a certain intensity of stimulation (Han, 1994; Liang et al., 2001; Huang, 2006). Over the last decade, an increasing number of studies on acupuncture using functional magnetic resonance imaging (fMRI) has explored instant effects with blood oxygenation level-dependent (BOLD) signal changes and sustained effects with resting-state network modulations (Qin et al., 2006, 2008; Dhond et al., 2007, 2008; Bai et al., 2009; Feng et al., 2011; Huang et al., 2012; Zhong et al., 2012; Jiang et al., 2013; You et al., 2013; Nierhaus et al., 2015). Common approaches for the analysis of resting-state fMRI data are seed-based correlation analysis and spatial independent component analysis (ICA). Dhond et al. (2008) used ICA on resting-state data and reported that the acupuncture point stimulation increased functional connectivity of the default mode network (DMN) to pain, affective and memory related regions, and also increased sensorimotor network (SMN) connectivity to pain-related brain regions. Qin et al. (2008) used seed-based correlation analysis to identify the amygdala-related network both after real and penetrating sham acupuncture. However, both methods are limited by *a priori* definitions of a “seed” or a “component of interest” for the analysis, thus, interesting associations may have been overlooked.

Recently, the functional connectome of the human brain has attracted increasing interest and graph theory based investigation of “network hubs” or functional structure property has been successfully implemented in several neuroimaging studies (Lohmann et al., 2010; Taubert et al., 2011). However, to our knowledge only a few studies have used this approach to explore the modulation of the entire brain’s functional connectome after acupuncture stimulation (Liu et al., 2010, 2011). Centrality mapping is a graph theory based connectivity analysis, which can be used to characterize one aspect of the whole-brain functional connectome. Independent of assumptions, every voxel is considered as a “seed” (node) and its connectivity to all other voxels is estimated. Based on this, a centrality value is determined that describes the impact of each voxel within the whole brain network. Thus, centrality mapping is a data driven approach that summarizes the connectome information for each voxel (Zuo et al., 2012).

We previously investigated instant acupuncture effects based on BOLD data derived from an event-related needle stimulation, as well as sustained effects using seed-based resting state connectivity (Nierhaus et al., 2015). Cerebral responses were evaluated after standardized needle stimulation on three different point locations on the right leg: one acupuncture point ST36 and two control points which are not acupuncture points. One of the control points was nearby ST36 in the same dermatome L5 (CP1), while the other was in a different dermatome L2 (CP2). Compared to control point stimulation, we found more pronounced activations in insula and secondary somatosensory cortex (S2), as well as a deactivation in precuneus during stimulation of ST36. In addition, S2 seed-based functional connectivity analysis showed increased connectivity of S2 to right precuneus after stimulation of ST36. These regions are well known pain-related areas and hint to a potential mechanism of pain modulation due to ST36 acupuncture stimulation (Nierhaus et al., 2015). However, the seed-based approach describes only the connectivity of one pre-defined region.

For this study, we re-analyzed the resting-state data with two centrality mapping analyses: degree centrality mapping (DCM) and eigenvector centrality mapping (ECM). While DCM is simply the sum of connectivity strength between all pairs of nodes (voxels), ECM uses the first eigenvector of the correlation matrix as the weight to summarize the connections of every voxel. Hence, DCM can be defined as the number of links which a node has, while ECM measures the influence of a node within a network [favors nodes that are connected to nodes that are themselves central within the network (Lohmann et al., 2010)]. A well-known example of an ECM application is Google’s PageRank algorithm. Thus, DCM measures more local centrality and ECM measures more global centrality (Zuo et al., 2012). Both DCM and ECM have been implemented in previous fMRI studies and significant as well as meaningful differences between different brain states or participants have been reported (Buckner et al., 2009; Lohmann et al., 2010; Fransson et al., 2011; Taubert et al., 2011). Based on our prior results, we wanted to evaluate whether these two centrality approaches also show differences when comparing needle stimulation at an acupuncture point with stimulation at two non-acupuncture control points.

## Materials and Methods

### Subjects

Twenty-two healthy subjects (11 male, mean age 26 years, range 21–32 years, right-handed) participated in the fMRI measurement as described in Nierhaus et al. (2015). Participants had no medical knowledge about acupuncture and all except one had never been treated with acupuncture before the study. The original study consisted of an EEG and an fMRI experiment. Eight subjects participated in both EEG and fMRI, thus were not completely naïve to acupuncture when performing the fMRI measurement. Participants were informed about the needle stimulation as follows: “…one acupuncture needle will be inserted in the muscle at three different points at the upper and lower leg….” All participants were right-handed and gave written informed consent to participate in the experiment according to the declaration of Helsinki. The ethics committee of Leipzig University approved the study (Nr. 214-09-28092009) and we registered the study at ClinicalTrials.gov (NCT01079689). Prior to participation, all subjects underwent a comprehensive neurological examination and confirmed they were not taking any acute or chronic medication. One female subject was excluded from the measurements because of vegetative side effects (severe sweating) during the stimulation on the acupuncture point.

### Experimental Procedure

**FIGURE 1:**
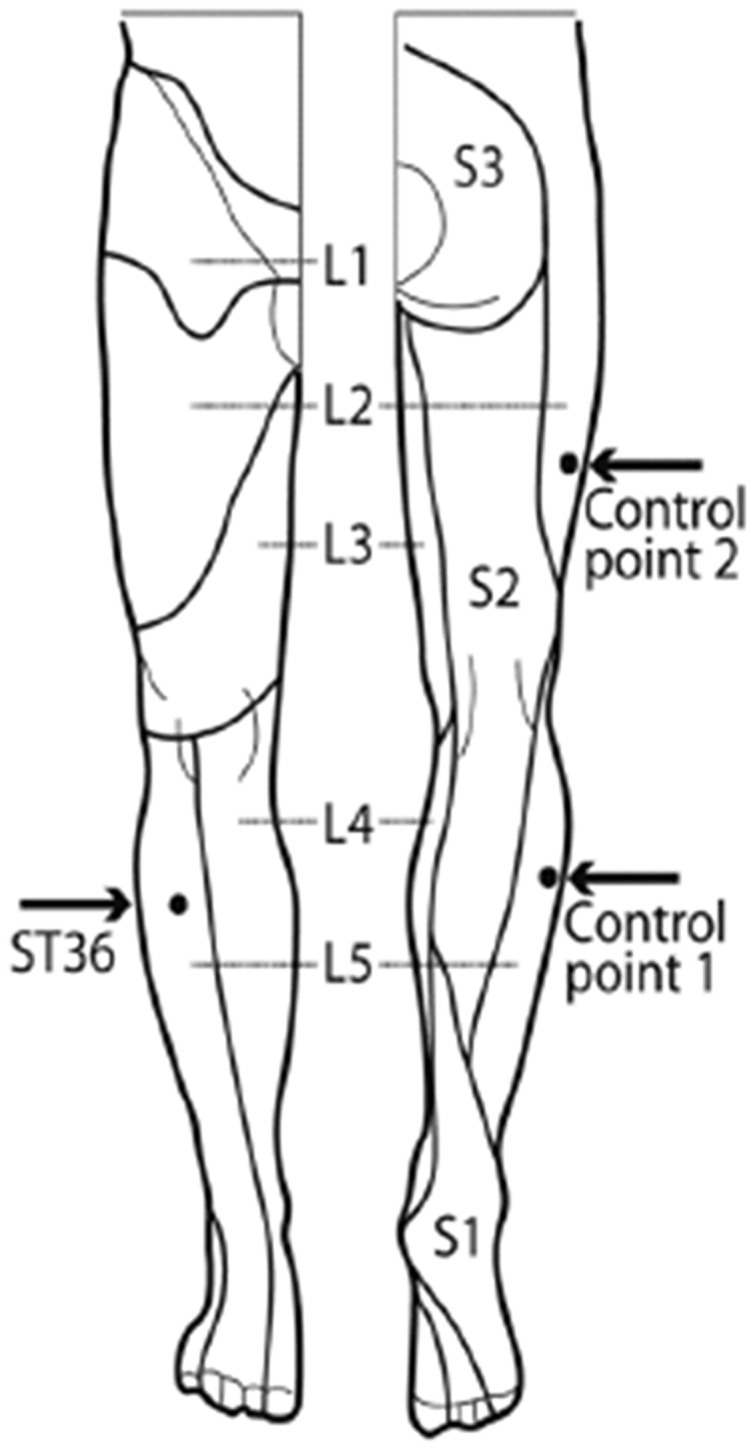
**Locations of acupuncture point ST36 and the control points on the right leg [view from the front and from the back, figure adapted (Drake et al., 2009)].** ST36 is located on the anterior aspect of the right leg, on the line connecting ST35 with ST41, 3 B-cun inferior to ST35. The location of ST36 belongs to the dermatome L5. Control point 1 (CP1) is located lateral to ST36, at the middle line between Bladder meridian and Gallbladder meridian, in the same dermatome L5. Control point 2 (CP2) is located 2 B-cun dorsally of GB31, the location of CP2 belongs to the dermatome L2 (Nierhaus et al., 2015).

Subjects were scanned and received the needle stimulation at the three points within one session. The acupuncture point was ST36 (Zusanli). Control point 1 (CP1) was in the same dermatome L5 as ST36, and control point 2 (CP2) was in the (different) dermatome L2 (**Figure 1**). The locations for the control points were carefully chosen after literature screening and a consensus process between one of the authors (FL) and a second expert of the Chengdu University of TCM (Prof Li Ying). The rational of the point selection can be found in previous publications (Nierhaus et al., 2015, 2016; Wong, 2016).

**FIGURE 2:**
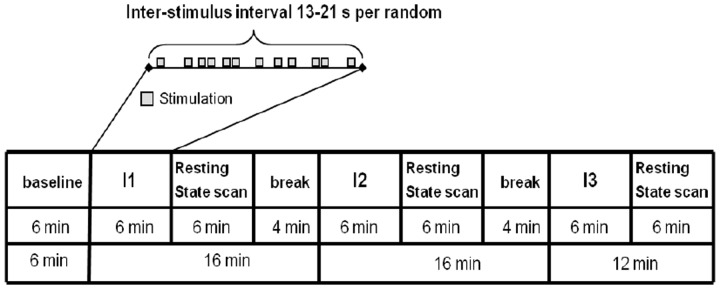
**The paradigm of the experiment included needle interventions and resting-state scans.** The upper part represents the needle stimulation sequence. The lower part represents the whole run of the experiment. Four resting-state scans including one baseline resting-state scan and three scans after needle stimulation were performed. For the Interventions I1/I2/I3 the sequence of the stimulated points (ST36/CP1/CP2) was randomized across participants (Nierhaus et al., 2015).

The experimental paradigm is shown in **Figure 2**. One resting-state scan was performed at the beginning as the baseline scan (RS_B), then three scans with needle stimulation of one point in an event-related design, each followed by a 6 min’ corresponding resting state scan (i.e., RS_ST36, RS_CP1, and RS_CP2). During scanning, subjects were told to remain in the supine position with open eyes and concentrate on the sensation caused by the needle stimulation. During the resting-state, participants were requested to keep calm and stay still with eyes open.

The penetrating needle stimulation was performed by an acupuncture physician with sterile, single use, individually wrapped needles (0.20 mm × 30 mm; titanium, DongBang, Acupuncture, Inc., Boryeong, Korea). The needle was first inserted 1–2 cm deep into the skin depending on the size of the muscle vertically on the right leg. Then the needle was manually manipulated according to the event-related design starting immediately after insertion. Auditory cues signaled the timing of the stimulation events to the acupuncturist via headphones. Each event consisted of 3 s stimulation from needle rotating 60–90/rpm and lift-thrusting 0.3–0.5 cm. The length of the inter-stimulus interval was randomized from 13 to 21 s. The needle was taken out after the 6 min event-related needle manipulation. Identical penetrating needle stimulation was performed on the three different point locations (**Figure 2**). The order of point locations was randomized.

### Data Acquisition

Data was acquired using a 3T Siemens Verio MRI System (Siemens Medical, Erlangen, Germany) equipped for echo planar imaging with a 12-channel head coil. fMRI images were acquired using an EPI sequence (30 axial slices, in-plane resolution is 3 mm × 3 mm, slice thickness = 4 mm, flip angle = 90°, gap = 1 mm, repetition time = 2000 ms, echo time = 30 ms). A structural image was also acquired for each participant, using a T1-weighted MPRAGE sequence (repetition time = 1200 ms, echo time = 5.65 ms, and flip angle = 19°, with elliptical sampling of *k* space, giving a voxel size of 1 mm × 1 mm × 1 mm). The subjects’ heads were immobilized by cushioned supports and they wore earplugs to attenuate MRI gradient noise throughout the experiment.

### Resting-State fMRI Data Analysis

The first ten volumes of each resting-state scan (RS_B, RS_ST36, RS_CP1, and RS_CP2) were removed to account for adaptation of the participant to scanner noise and environment. Slice timing, head motion correction and spatial normalization to MNI152 space were performed by SPM8^1^. T1 images were segmented into gray matter, whiter matter and cerebral spinal fluid (CSF). For spatial normalization, the gray matter image was co-registered to the MNI152 template and a transformation matrix was created. The functional images were co-registered to the gray matter image, and then the transformation matrix was used for spatial normalization to the MNI152 space with the voxel size 3 mm × 3 mm × 3 mm. The differences of head motion across resting-state scans were examined by comparing the averaged frame-wise displacement (mean FD) using BRAMILA tools (Power et al., 2012)^2^. The toolbox REST^3^ was used for temporal band-pass filtering (0.01–0.08 Hz) and removal of linear trends (Fox et al., 2009). The global mean signal was not regressed out since this step might affect the correlation between time courses (Buckner et al., 2009; Lohmann et al., 2010; Fransson et al., 2011; Taubert et al., 2011). DPABI toolbox (toolbox for Data Processing & Analysis of Brain Imaging^4^) was used to apply the CompCor method (Behzadi et al., 2007). This method applies the combined CSF/white matter mask on the resting-state data and performs a principal component analysis to extract associated variance. The first five principal components from the CompCor analysis and six head motion parameters from the motion correction were used as nuisance signals to regress out associated variance. No spatial smoothing was applied before the centrality analysis, as this could generate artificially high correlation coefficients (Zuo et al., 2012). A gray matter mask [around 39429 voxels, more details in Nierhaus et al. (2015)] derived from the segmented T1 images was used for the centrality analysis. For each individual resting-state scan, the eigenvector centrality map and degree centrality map was generated by fastECM, which provides a more efficient way to perform the centrality analysis without calculating the voxel-wise correlation matrix (Wink et al., 2012). Z-standard transform (i.e., for each voxel, subtract the mean value of the whole brain then divide by the standard deviation of the whole brain) and 6 mm FWHM smoothing were performed on the individual DCM and ECM maps (Zuo et al., 2012; Yan et al., 2013). A within subjects ANOVA on the ECM/DCM maps of all four resting-state scans as well as age and gender as covariates were performed. The comparisons to baseline (RS_ST36-RS_B, RS_CP1-RS_B, and RS_CP2-RS_B, see Supplementary Material), the inter-points comparisons (RS_ST36-RS_CP1, RS_ST36-RS_CP2, and RS_CP1-RS_CP2), and the conjunction maps were generated. Conjunction of “RS_ST36-RS_CP1 and RS_ST36-RS_CP2” was calculated to compare the acupuncture point and the control points, while conjunction of “RS_ST36-RS_CP2 and RS_CP1-RS_CP2” was calculated to compare the different dermatomes (L5 vs. L2). All statistical maps (i.e., T-map) were corrected for multiple comparison to the alpha-level *p*_corr_ < 0.05 using AlphaSim in AFNI [Version 16.2.12, (Cox, 1996)] as follows: (1) The smoothing parameters were estimated using the 3dFWHMx function on the 6 mm (FWHM) smoothed, pre-processed fMRI time courses and averaged across all participants’ resting-state sessions; (2) These smoothing parameters, the voxel-level *p*-value (*p*_vox_ < 0.01), and the gray matter mask (39429 voxels) were used as the input for the 3dClustSim function, resulting in a cluster size of 2052 mm^3^ to reach significance (*p*_corr_ < 0.05); (3) The combination of voxel-level *p*-value (<0.01) and cluster size (2052 mm^3^) was then applied to threshold each statistical map. Regions which were detected in the conjunction analysis were selected as ROI. The average and standard error of mean (SEM) of the *z*-value within each ROI were calculated for each resting-state scan and both centrality approaches.

Dice’s coefficient (DC, from 0 to 100%, with 100% meaning that two maps are completely overlapping with one another) was employed to estimate the overlap ratio between the corrected statistical maps of post-stimulation centrality of the two different centrality analyses (ECM vs. DCM) (Dice, 1945).

## Results

### Head Motion

There was no significant difference in head motion (mean FD) across all three post-stimulation resting-state scans (*p* = 0.7495, one-way ANOVA). There was no significant difference between scans: RS_ST36 vs. RS_CP1, *p* = 0.2610; RS_ST36 vs. RS_CP2, *p* = 0.5931; RS_CP1 vs. RS_CP2, *p* = 0.2429.

### Post-effects of Needle Stimulation of all Three Points against each Other

**FIGURE 3:**
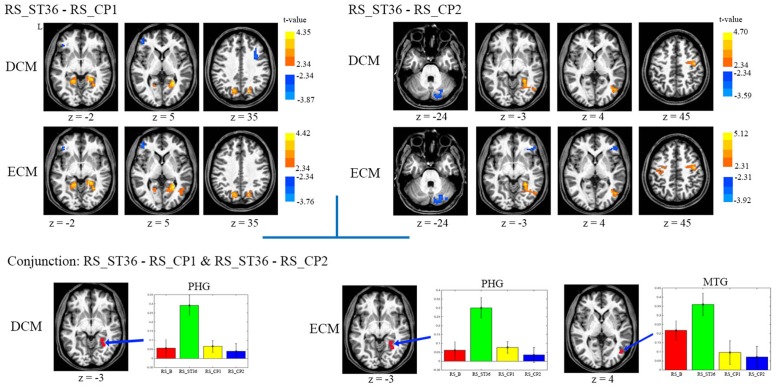
**The centrality changes of the post-stimulation of all three points against each other.** L means the left hemisphere. The warm color means increased centrality and the cold color means decreased centrality. All images were in the Talairach space. *P* < 0.05, corrected. The average centrality value (mean ± SEM) of the related centrality measures from each resting-state scan within the selected ROIs which were detected in the conjunction analysis were displayed. No significant result was found in the comparison between CP1 and CP2 for both ECM and DCM analysis. Abbreviations: PHG, parahippocampus gyrus; MTG, middle temporal gyrus.

The comparison between ST36 and CP1 revealed higher centrality for ST36 in the right parahippocampal gyrus, bilateral posterior cingulate cortex/precuneus, lower centrality for ST36 in the left dorsolateral prefrontal cortex by both ECM and DCM analyses (**Table 1**, **Figure 3**). Lower centrality for ST36 in the right middle frontal cortex was only detected by DCM, and higher centrality in right middle temporal gyrus only by ECM.

**Table 1:** Post-stimulation centrality changes of all three points against each other (*P* < 0.05, corrected) and the results of the conjunction analysis.

	Area	Left/Right	BA	*x. y. z*	*T-*value	*p-*value	Volume (mm^3^)
**DCM**							
RS_ST36-RS_CP1	Parahippocampal gyrus	R	30	28, -53, 1	4,35	4,25E-04	7344
	Posterior cingulate/Precuneus	L	31	-17, -64, 25	4,30	4,78E-04	4536
	Dorsolateral prefrontal cortex	R	6	39, 8, 41	-3,65	2,04E-03	2160
		L	10	-42, 45, 12	-3,87	1,24E-03	2133
RS_ST36- RS_CP2	Parahippocampal	R	19	25, -44, -4	4,70	1,97E-04	5940
	gyrus/Middle temporal gyrus						
	Pre/Postcentral gyrus (M1/S1)	R	6	39, -11, 41	3,93	1,09E-03	2835
	Declive	R	/	19, -79, -20	-3,59	2,31E-03	2133
Conjunction	Parahippocampal gyrus	R	19	/	/	/	1026
**ECM**							
RS_ST36-RS_CP1	Parahippocampal gyrus	R	30	28, -53, 1	4,42	3,65E-04	11097
	Posterior cingulate/Precuneus	L	31	-17, -64, 25	4,19	6,09E-04	5670
	Dorsolateral prefrontal cortex	L	10	-42, 45, 12	-3,76	1,58E-03	2322
RS_ST36- RS_CP2	Parahippocampal gyrus/Middle	R	19	25, -44, -4	5,12	7,78E-05	7317
	temporal gyrus						
	Declive	R	/	19, -79, -20	-3,92	1,11E-03	4077
	Pre/Postcentral gyrus (M1/S1)	R	6	39, -11, 41	4,12	7,10E-04	3537
		L	4	-34, -13, 46	3,99	9,58E-04	2970
	Orbital frontal cortex	R	45	47, 36, 1	-3,29	4,53E-03	2214
Conjunction	Parahippocampal gyrus	R	19	/	/	/	1296
	Middle temporal gyrus	R	37	/	/	/	1161


**x, y, z* is in Talairach space. Displayed are voxels of maximal significance. If the activated area crosses the midline, only the side of the highest value is displayed. *T*- and *p-*value is on the voxel-level. No significant result was found in the comparison between CP1 and CP2 for both ECM and DCM analysis.*

Compared to CP2, ST36 showed higher centrality in the right parahippocampal gyrus, right sensorimotor cortex and right middle temporal gyrus, and lower centrality in cerebellum by ECM and DCM. Lower centrality for ST36 in the right inferior frontal gyrus and higher centrality for ST36 in the left sensorimotor cortex was only seen in ECM (**Table 1**, **Figure 3**).

There was no significant result in the comparison between CP1 and CP2 for both ECM and DCM analysis.

### Conjunction Analysis

In order to identify brain areas which were specifically modulated due to the acupuncture point and dermatome effect, respectively, we performed two conjunction analyses. The comparison of acupuncture point vs. control points (conjunction of RS_ST36-RS_CP1 and RS_ST36-RS_CP2) showed common positive differences (higher centrality for ST36) in parahippocampal gyrus by both ECM and DCM. Another common region with positive differences was found in middle temporal gyrus, but only by ECM (**Figure 3**). Comparing the effects after stimulation in two different dermatomes L5 and L2 (conjunction of RS_ST36-RS_CP2 and RS_CP1-RS_CP2) revealed no overlapping regions with common differences in centrality.

The mean values for the ROIs selected from the conjunction analysis are shown in **Figure 3**. PHG showed significantly higher centrality for RS_ST36, in contrast to lower centrality for the other three resting-state scans (baseline and the two control points) in both, ECM and DCM. The cluster in middle temporal gyrus (only for ECM) also showed higher centrality for RS_ST36.

### Dice Coefficient Analysis

The overlap of two different methods was also calculated (**Table 2**). DCM and ECM showed an overlap of 76.62 and 69.80% for the point comparisons RS_ST36-RS_CP1 and RS_ST36-RS_CP2, respectively. The overlap for the baseline comparisons is shown in the Supplementary Material.

**Table 2:** Dice coefficient analysis: Overlap ratio (percentage) of ECM and DCM results.

	All	Positive	Negative
RS_ST36-RS_CP1	76.72	80.87	58.78
RS_ST36-RS_CP2	69.80	76.94	50.64


*Positive/negative means the positive/negative differences in the statistical maps.*

## Discussion

We applied centrality mapping analysis to previously published data from an acupuncture neuroimaging study (Nierhaus et al., 2015). Employing different types of analyses, we evaluated instant and sustained acupuncture effects after event-related needle stimulation on one acupuncture point (ST36) in comparison to needle stimulation of two control points (non-acupuncture points, CP1 in the same dermatome, CP2 in a different dermatome). While in our previous analysis, we had evaluated instant BOLD effects and S2-seed-based resting state connectivity (Nierhaus et al., 2015), here, we used ECM and DCM to add new insights in the sustained acupuncture effect on both global and local connectivity (centrality) within the whole-brain.

To identify centrality differences between stimulation at ST36 and both control points, we performed a conjunction analysis of the point comparisons between ST36-CP1 and ST36-CP2, and we furthermore performed a conjunction analysis of the point comparisons between CP2-CP1 and CP2-ST36. If the main differential effects of the three stimulation sites are due to the different dermatomes then one would expect a strong discriminating effect of CP2 when comparing it to the two other sites. If the main differential effects, however, are due to stimulating the acupuncture point, one would expect the strongest discriminating effect when comparing ST36 to the other two stimulation sites. Our results demonstrate that the latter was the case: The conjunction ST36-CP1/ST36-CP2 showed overlapping clusters of increased centrality for ST36 in right parahippocampal gyrus, by both DCM and ECM. This conjunction analysis also showed a common cluster of increased centrality for ST36 in right middle temporal gyrus, but only in the ECM analysis. We performed no conjunction for the dermatome comparison (CP2 vs. ST36 and CP2 vs. CP1), because already the control point comparison (CP1 vs. CP2) showed no significant clusters. Thus, in accordance with our previous analysis (Nierhaus et al., 2015), these results suggest differential processing of acupuncture point stimulation compared to stimulation of non-acupuncture control points, now from the perspective of the whole-brain network. Parahippocampal gyrus and middle temporal gyrus might be important hubs affected by stimulation of an acupuncture point and these results provide further hints toward potential mechanisms of pain modulation by acupuncture stimulation.

Previous studies already indicated that parahippocampal gyrus might contribute to the effect of acupuncture of an acupuncture point (Huang et al., 2012; Chae et al., 2013). The BOLD signal in parahippocampal gyrus was found mainly deactivated during acupuncture stimulation in several previous neuroimaging studies (Wu et al., 1999, 2002; Hui et al., 2005; Napadow et al., 2005; Yan et al., 2005; Wang et al., 2007; Fang et al., 2009), and also in our previous analysis for all three points during stimulation (Nierhaus et al., 2015). Deactivation in parahippocampal gyrus was found to be related to acupuncture sensation, such as pressure, numbness, heaviness, and fullness (Wang et al., 2013). According to Jiang et al. (2014) the level of cerebral blood flow in parahippocampal gyrus is negatively correlated to analgesia after transcutaneous electric stimulation of an acupuncture point, suggesting acupuncture might inhibit pain by inhibiting the pain signal in the parahippocampal gyrus at a later stage. Moreover, recent research found that parahippocampal gyrus is related to the experience of chronic pain and anxiety, and structural changes in parahippocampal gyrus were found in chronic pain patients (Smallwood et al., 2013). When accompanying anxiety increases, the pain worsens, resulting in activation in parahippocampal gyrus (Ploghaus et al., 2001). In a study by Vachon-Presseau et al. (2013) chronic pain was associated with maladaptive stress and was reflected in hippocampal structural differences.

Beside its participation in cognitive processes such as language and multimodal semantic processing (Tranel et al., 1997; Chao et al., 1999; Cabeza and Nyberg, 2000; Visser et al., 2012). Middle temporal gyrus has previously been shown to be related to pain and effects of acupuncture. Recent studies found significantly reduced gray matter volumes in middle temporal gyrus in chronic pain patients, e.g., lower back pain (Luchtmann et al., 2014), cluster headache (Absinta et al., 2012), migraine (Rocca et al., 2006), and cerebral post stroke pain (Krause et al., 2016). However, the role of middle temporal gyrus involved in chronic pain processing is still unclear (Krause et al., 2016). According to a study by Yan et al. (2005) the BOLD signal in middle temporal gyrus increased when acupuncture points were stimulated. Also in our previous analyses middle temporal gyrus was activated during stimulation, but for ST36 and CP1 in the same dermatome, whereas deactivated for CP2 in a more proximal different dermatome.

Moreover, both parahippocampal gyrus and middle temporal gyrus can be linked to the DMN (Greicius et al., 2003; Laird et al., 2009; Ward et al., 2014) (see also supplemental analysis). The DMN might be the primary network affected by chronic pain (Farmer et al., 2012; Baliki et al., 2014). Dhond et al. (2008) showed increased DMN connectivity with limbic antinociceptive (anterior cingulated cortex, periaqueductal gray), affective (amygdala, anterior cingulated cortex), and memory (hippocampal formation, middle temporal gyrus) related brain regions following acupuncture, but not sham. The DMN was deactivated during acupuncture stimulation (Bai et al., 2009; Napadow et al., 2013), but not when acupuncture was associated with sharp pain (Hui et al., 2009). For chronic pain conditions, such as chronic lower back pain and migraine, the pain relief was correlated with DMN alteration after acupuncture treatments (Li et al., 2014; Zhao et al., 2014). Also DMN modulation was found after acupuncture stimulation in mental disorders, e.g., major depressive disorder (Deng et al., 2016) and Alzheimer’s disease (Liang et al., 2014). In another study parahippocampal gyrus was shown to be a connecting hub between the DMN and the temporal lobe memory system (Ward et al., 2014). Several studies show that the DMN activity/connectivity is related to memory-based processing (Buckner et al., 2008; Vatansever et al., 2015), and is modulated in the presence of pain, especially chronic pain (Baliki et al., 2014).

Together with our previous results (Nierhaus et al., 2015), our data point to a possible mechanism that explains the pain relieving effectiveness of acupuncture, especially in chronic pain: we found acupuncture related (i) activation of S2 and insula, which are assumed to contribute to the experience of pain (Craig, 2009), (ii) strengthened functional connectivity between S2 and precuneus which seems to be involved in the assessment and integration of pain (Goffaux et al., 2014), and (iii) increased whole brain functional connectivity in parahippocampal gyrus and middle temporal gyrus, which are both involved in DMN activity and might modulate the memory for pain.

Possible limitations of our study should be discussed: Regarding the experimental setup, we tried to come as close as possible to a double-blind study (Nierhaus et al., 2015) but of course the person applying the acupuncture stimulation knew about the “meaning” of the different points. Regarding data analysis, although the centrality mapping could give new insights into imaging data it can be limited by its voxel-wise temporal correlation analysis which may produce spurious correlations between voxels. For instance, a spurious correlation may be introduced between regions if this correlation is modulated by other regions. To avoid this, partial correlation analysis might be included into centrality mapping methods in future studies (Smith et al., 2011). Moreover, centrality mapping allows the characterization of only one aspect of the whole-brain functional connectome, i.e., the summary of connections of every voxel. For the broader picture, other graph theory based methods should be used in future research. Possible ‘carry-over effects’ between the different interventions might be an additional limitation. However, by randomizing the order of point locations all three interventions should be comparably affected by this phenomenon in our study. To avoid carry-over effects, the different interventions might be separated over a longer period of time, e.g., 24 h or even more. But the allocation of the different interventions to separate days would require a more complex experimental setup. Moreover, the duration of the sustained effect of acupuncture is not clear and our 6-min resting-state measurements might cover only its initial phase. Longer measurement periods would be necessary to cover long-term effects and to analyse the variation of functional connectivity in time.

To our knowledge, this is the first study applying ECM for the investigation of the sustained effect of acupuncture stimulation. Employing ECM and DCM, we investigated global and local functional connectivity of the acupuncture effect and compared both methods. As shown by a high Dice coefficient (around 75%) most of the results were similar between the ECM and the DCM analysis for all three stimulation points, maybe because the whole brain centrality intensity calculated by these two approaches are both highly positively correlated (Zuo et al., 2012). However, we found also differences when using the two approaches, e.g., the right frontal cortex was detected by DCM but not by ECM in the comparison between RS_ST36 and RS_CP1. Both methods’ sensitivity seems to be different and the sensitivity might depend on the role of the respective brain areas within the whole-brain connectome (Gottlich et al., 2014). Previous studies (Liu et al., 2010, 2011; Ren et al., 2010; Fang et al., 2012) had applied graph theory analysis to investigate the post-needling resting state in healthy subjects. Ren et al. (2010) observed distinct signal changes in different brain regions within a group of ROIs between manual manipulations on three different acupuncture points by graph theory analysis. For the acupuncture point GB37 (located on the leg) they found PCC to show a larger degree of connectivity following stimulation. Liu et al. (2010, 2011) also used graph theory to investigate the whole-brain functional connectivity between identical manual stimulation on ST36 and one nearby control point. Significant degree differences were found in limbic areas, thalamus, brain stem, prefrontal cortices, temporal cortices, and cerebellum. Fang et al. (2012) measured local and distant degree centrality changes after electro-acupuncture stimulation on two acupuncture points separately, and found that ST36 led to stronger degree centrality changes than CV4 in the limbic-paralimbic-neocortical network. The analysis methods used in these studies are similar to DCM in our current study, however, the t-maps of them were all uncorrected. In our study, we additionally used ECM analysis, and all the t-maps were statistically corrected. Our findings correspond well with the significant differences between ST36 and control points that were already described by Liu et al. (2010, 2011) and Fang et al. (2012).

## Conclusion

According to our data-driven methods, centrality changes in parahippocampal gyrus and middle temporal gyrus hint to possible specific differences of the sustained effect between the acupuncture point ST36 and two control points. The stronger integration of parahippocampal gyrus and middle temporal gyrus within the whole-brain network after stimulation of an acupuncture point might be a potential contributor to pain modulation. We think both centrality mapping analysis ECM and DCM could be valuable data-driven tools with add-on value for future imaging studies investigating the effect of acupuncture.

## Author Contributions

Conceived and designed the experiments: TN, WH, DP, CW, AV, VN, and FL. Performed the trial: TN, WH, and DP. Analyzed the data: XL. Discussed the data: TN, WH, DP, XL, CW, AV, VN, BP, and FL. Wrote the first draft of the paper: XL, WH, TN, and DP. Revised the paper and approved the final version: TN, WH, DP, XL, CW, AV, VN, BP, and FL.

## Conflict of Interest Statement

The authors declare that the research was conducted in the absence of any commercial or financial relationships that could be construed as a potential conflict of interest.

## Footnotes

1 www.fil.ion.ucl.ac.uk/spm/

2 http://becs.aalto.fi/~eglerean/bramila.html

3 www.restfmri.net

4 http://rfmri.org/dpabi
