# Supplementary material for: Sustained Effects of Acupuncture Stimulation Investigated with Centrality Mapping Analysis
Source: Front Hum Neurosci. 2016 Oct 18;10:510. doi: 10.3389/fnhum.2016.00510 (PMC5067410; doi:10.3389/fnhum.2016.00510)
Supplement: Supplementary file 1 [file Data_Sheet_1.DOCX]

**Supplemental Material**

## Post-effects of needle stimulation against baseline

After stimulation of ST36, both ECM and DCM revealed decreased centrality in the left dorsolateral prefrontal cortex (dlPFC), as well as increased centrality in bilateral sensorimotor cortex, parahippocampal gyrus (PHG), the right posterior cingulate cortex (PCC)/precuneus and the right middle temporal gyrus (MTG) (supTable 1, supFig. 1). Increased centrality in the right secondary somatosensory cortex (S2) were only found by DCM.

After stimulation of CP1 both DCM and ECM revealed increased centrality in the left S1/M1 (supTable 1, supFig. 1).

After stimulation of CP2 both methods revealed increased centrality in PCC/precuneus (supTable 1, supFig. 1).

**
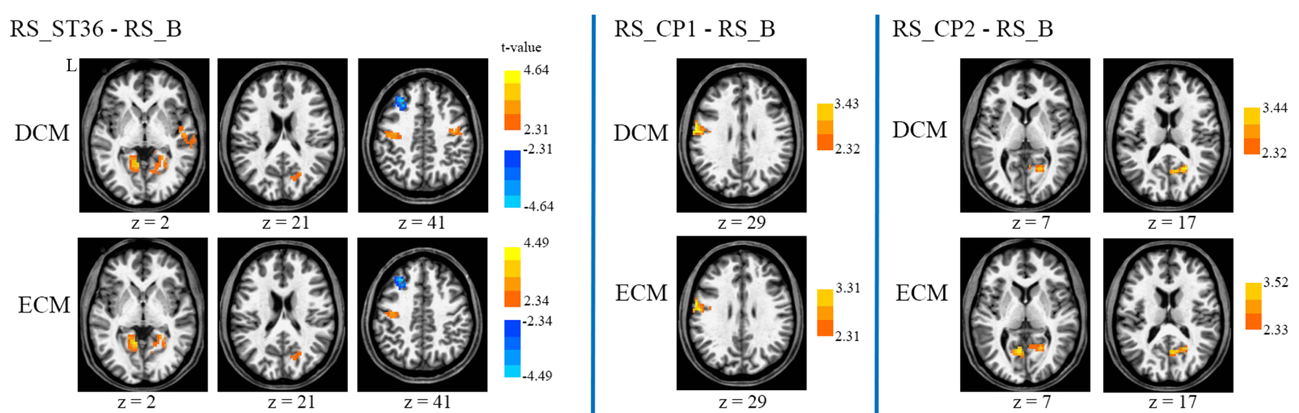
**

**Supplemental Figure 1.** Centrality changes post-stimulation of ST36 and two control points against baseline. R means the right hemisphere. The warm colour means increased centrality and the cold colour means decreased centrality. The axial slices were selected to present the regions which were listed in the related tables. All images were in the Talairach space. P< 0.05, corrected.

## Seed-based functional connectivity analysis of the conjunction areas (PHG and MTG)

We performed this additional analysis to evaluate whether the ‘conjunction areas’ were connected to the default-mode-network.

The two brain areas from the ECM conjunction analysis (PHG and MTG, see manuscript figure 3 and table 1) were implemented as the ROIs for a seed-based functional connectivity analysis on the first resting-state session (RS_B) across participants. Then all functional connectivity maps were Fisher’s z transformed and a one sample t-test was performed on all functional connectivity maps.

The functional connectivity maps of both ROIs presented positive correlations to the major hubs of the DMN, i.e. posterior cingulate cortex / precuneus and medial prefrontal cortex (supplementary fig. 2).


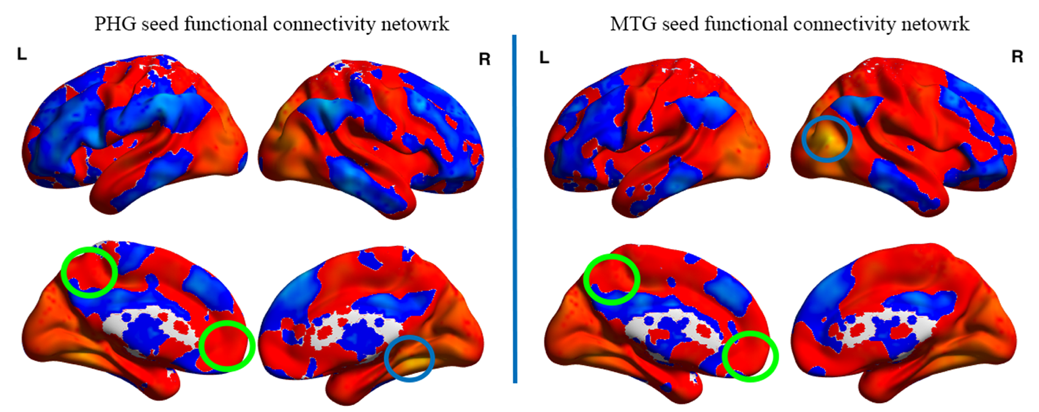


**Supplemental Figure 2.** The unthresholded functional connectivity maps of the two ROIs (blue circled). The green circles indicate the major hubs of the default mode network, i.e. posterior cingulate cortex / precuneus and medial prefrontal cortex. The warm colors indicate positive correlations, the cold colors indicate negative correlations to the ROI.

**Supplemental Table 1**: Post-stimulation centrality changes of ST36 and two control points against baseline. (P<0.05, corrected), x, y, z is in Talairach space. Displayed are voxels of maximal significance. If the activated area crosses the midline, only the side of the highest value is displayed. T and p value is on the voxel-level.

| **DCM** | **Area** | **Left/Right** | **BA** | **x, y, z** | **T value** | **p value** | **Volume**  **(mm^3^)** |
| --- | --- | --- | --- | --- | --- | --- | --- |
| **RS_ST36-RS_B** | Posterior Cingulate/Precuneus | R | 30 | 19, -56, 12 | 4,64 | 2,24E-04 | 8370 |
|  | Pre/Postcentral Gyrus (M1/S1) | L | 6 | -39, -8, 33 | 4,04 | 8,56E-04 | 3807 |
|  |  | R | 6 | 36, -13, 36 | 3,73 | 1,70E-03 | 2268 |
|  | Dorsolateral Prefrontal Cortex | L | 8 | -28, 28, 41 | -4,51 | 2,98E-04 | 3024 |
|  | Parahippocampal Gyrus | L | 19 | -14, -50, -2 | 4,41 | 3,71E-04 | 2781 |
| **RS_CP1- RS_B** | Precentral Gyrus | L | 6 | -56, -3, 30 | 3,43 | 3,29E-03 | 3456 |
| **RS_CP2- RS_B** | Posterior Cingulate/Precuneus | R | 30 | 19, -56, 14 | 3,44 | 3,27E-03 | 3348 |

| **ECM** | **Area** | **Left/Right** | **BA** | **x, y, z** | **T value** | **p value** | **Volume (mm^3^)** |
| --- | --- | --- | --- | --- | --- | --- | --- |
| **RS_ST36-RS_B** | Posterior Cingulate/Precuneus | R | 30 | 19, -56, 12 | 4,49 | 3,16E-04 | 7695 |
|  | Pre/Postcentral gyrus (M1/S1) | L | 6 | -39, -8, 33 | 3,93 | 1,09E-03 | 2808 |
|  | Parahippocampal gyrus | L | 19 | -14, -50, -2 | 4,25 | 5,30E-04 | 2673 |
|  | Dorsolateral Prefrontal Cortex | L | 8 | -28, 28, 41 | -4,36 | 4,16E-04 | 2457 |
| **RS_CP1- RS_B** | Precentral Gyrus (M1) | L | 6 | -56, -3, 30 | 3,31 | 4,33E-03 | 2700 |
| **RS_CP2- RS_B** | Posterior Cingulate /Precuneus | R | 30 | 6, -59, 14 | 3,52 | 2,70E-03 | 3267 |
|  |  | L | 30 | -6, -59, 6 | 3,46 | 3,08E-03 | 2295 |

**Supplemental Table 2: Dice coefficient analysis:** Overlap ratio (percentage) of ECM and DCM results. Positive/negative means the positive/negative differences in the statistical maps.

|  |  | **All** | **positive** | **negative** |
| --- | --- | --- | --- | --- |
| **RS_ST36-RS_B** |  | 80.45 | 78.90 | 89.66 |
| **RS_CP1-RS_B** |  | 84.21 | 84.21 | / |
| **RS_CP2-RS_B** |  | 66.06 | 76.94 | / |
